# Epidermal Growth Factor Receptor Plays an Anabolic Role in Bone Metabolism In Vivo

**DOI:** 10.1002/jbmr.295

**Published:** 2010-11-18

**Authors:** Xianrong Zhang, Joseph Tamasi, Xin Lu, Ji Zhu, Haiyan Chen, Xiaoyan Tian, Tang-Cheng Lee, David W Threadgill, Barbara E Kream, Yibin Kang, Nicola C Partridge, Ling Qin

**Affiliations:** 1 Department of Orthopaedic Surgery, School of Medicine, University of PennsylvaniaPhiladelphia, PA, USA; 2 Department of Physiology and Biophysics, University of Medicine and Dentistry of New JerseyPiscataway, NJ, USA; 3 Department of Molecular Biology, Princeton UniversityPrinceton, NJ, USA; 4 Division of Radiobiology, Department of Radiology, School of Medicine, University of UtahSalt Lake City, UT, USA; 5 Department of Genetics, University of North CarolinaChapel Hill, NC, USA; 6 Department of Medicine, University of Connecticut Health CenterFarmington, CT, USA; 7 Department of Basic Science and Craniofacial Biology, New York University, College of DentistryNew York, NY, USA

**Keywords:** EPIDERMAL GROWTH FACTOR RECEPTOR, ANIMAL MODELS, BONE MASS, SKELETAL PHENOTYPE, OSTEOBLAST

## Abstract

While the epidermal growth factor receptor (EGFR)–mediated signaling pathway has been shown to have vital roles in many developmental and pathologic processes, its functions in the development and homeostasis of the skeletal system has been poorly defined. To address its in vivo role, we constructed transgenic and pharmacologic mouse models and used peripheral quantitative computed tomography (pQCT), micro–computed tomography (µCT) and histomorphometry to analyze their trabecular and cortical bone phenotypes. We initially deleted the EGFR in preosteoblasts/osteoblasts using a *Cre*/*loxP* system (*Col-Cre Egfr*^*f/f*^), but no bone phenotype was observed because of incomplete deletion of the *Egfr* genomic locus. To further reduce the remaining osteoblastic EGFR activity, we introduced an EGFR dominant-negative allele, *Wa5*, and generated *Col-Cre Egfr*^*Wa5/f*^ mice. At 3 and 7 months of age, both male and female mice exhibited a remarkable decrease in tibial trabecular bone mass with abnormalities in trabecular number and thickness. Histologic analyses revealed decreases in osteoblast number and mineralization activity and an increase in osteoclast number. Significant increases in trabecular pattern factor and structural model index indicate that trabecular microarchitecture was altered. The femurs of these mice were shorter and smaller with reduced cortical area and periosteal perimeter. Moreover, colony-forming unit–fibroblast (CFU-F) assay indicates that these mice had fewer bone marrow mesenchymal stem cells and committed progenitors. Similarly, administration of an EGFR inhibitor into wild-type mice caused a significant reduction in trabecular bone volume. In contrast, *Egfr*^*Dsk5/+*^ mice with a constitutively active EGFR allele displayed increases in trabecular and cortical bone content. Taken together, these data demonstrate that the EGFR signaling pathway is an important bone regulator and that it primarily plays an anabolic role in bone metabolism. © 2011 American Society for Bone and Mineral Research.

## Introduction

The epidermal growth factor receptor (EGFR) is a 170-kDa glycoprotein on the cell surface of a variety of cell types and is characterized by its ligand-dependent tyrosine kinase activity. The EGFR, also known as Her1 or ErbB1, is closely related to three other receptors, Her2 (ErbB2), Her3 (ErbB3), and Her4 (ErbB4). These receptors are composed of an extracellular ligand–binding domain with cystein-rich regions, a transmembrane domain, and an intracellular domain with tyrosine kinase activity. EGFR binds to a family of EGF-like ligands, including EGF, amphiregulin, and transforming growth factor α (TGF-α), heparin binding EGF (HB-EGF), betacellulin (BTC), and epiregulin. After ligand binding to the extracellular domain, the EGFRs are activated by homo- or heterodimerization with auto- and transphosphorylation on tyrosine residues at the intracellular domain, and then a variety of signaling pathways, such as Ras-Raf-MAP-kinase and PI-3-kinase-Akt, are activated to influence cell behaviors, such as proliferation, differentiation, apoptosis, and migration (reviewed in ref. 1). Owing to its critical role in tumorigenesis, two classes of drugs, monoclonal antibodies (mAbs) and tyrosine kinase inhibitors (TKIs), have been developed to target this receptor. While mAbs target the extracellular ligand–binding domain of EGFR and promote its internalization, TKIs block EGFR activity by competing with adenosine triphosphate (ATP) for binding to the receptor's kinase pocket. Three TKIs, gefitinib from AstraZeneca, erlotinib from OSI Pharmaceuticals, and lapatinib from GlaxoSmithKline, have received regulatory approval for use in cancer patients.(2)

While the EGFR-mediated signaling pathway has been shown to have a vital role in a variety of developmental and pathologic processes, its function in the development and homeostasis of the skeletal system has been poorly defined. Over the past several years, research from our laboratory and others suggests that EGFR signaling plays an important role in bone metabolism by affecting both bone formation and bone resorption. EGF and HB-EGF are potent mitogens for bone marrow stromal stem cells, the progenitor cells for osteoblasts.(3,4) Amphiregulin strongly stimulates the proliferation of preosteoblastic cells.(5) However, all EGF-like ligands greatly inhibit osteoblast differentiation in an EGFR-dependent pathway.(5,6) Moreover, they suppress gene expression of osteoblastic markers, such as alkaline phosphatase, bone sialoprotein (BSP), and osteocalcin, and the osteoblastic-specific transcription factors Runx2 and osterix.(6)

EGFR signaling also participates in bone resorption. EGF and TGF-α have the ability to strongly stimulate bone resorption in cultured fetal rat long bones, newborn mouse calvarial cultures, and long-term human marrow cultures,(7–9), suggesting that these growth factors regulate osteoclastogenesis and bone resorption. Previous studies in our laboratory delineated the molecular mechanism of stimulation of bone resorption by EGF-like ligands.(10) We found that EGF-like ligands stimulate osteoclastogenesis in an osteoblast/osteoclast coculture by decreasing osteoblastic expression of osteoprotegerin (OPG), a decoy receptor for the osteoclast determination factor, receptor activator for nuclear factor κβ ligand (RANKL), and increasing osteoblastic expression of monocyte chemoattractant protein 1 (MCP-1) but having no effect on RANKL expression.

EGFR deficiency leads to early lethality at midgestation, birth, or within 20 postnatal days depending on genetic background owing to severe developmental abnormalities in placental, neural, and epithelial tissues.(11–13) A few surviving *Egfr* null pups display craniofacial alterations and cleft palate.(14) At birth, *Egfr* null mice have delayed primary ossification of the cartilage anlage, and trabecular bone formation is impaired.(15) Mice humanized for EGFR (the endogenous mouse *Egfr* gene was replaced by human *EGFR* cDNA) exhibit low EGFR activity in bone.(16) They are growth retarded compared with wild-type siblings, but no overt bone remodeling defects were observed at birth. The most dramatic bone abnormality noted in these and *Egfr*^*–/–*^ mice is the greatly enlarged hypertrophic chondrocyte zone in the growth plate, suggesting that EGFR may play a role in chondrocyte terminal differentiation. By contrast, EGF-like ligand knockout mice are viable and fertile, implying an overlapping and compensating function among ligands. We have shown that amphiregulin knockout mice have significantly less tibial trabecular bone than wild-type siblings.(5) Moreover, overexpression of BTC ubiquitously results in a high cortical bone mass phenotype that is EGFR-dependent.(17)

In order to investigate the physiologic role of EGFR in bone development and remodeling in adult animals, we constructed three transgenic and pharmacologic mouse models with modified EGFR activity and performed a detailed analysis of their trabecular and cortical bone phenotypes in both sexes at different ages. We conclude from our data that the EGFR signaling pathway is an important bone regulator and that it primarily plays an anabolic role in bone metabolism.

## Materials and Methods

### Transgenic mouse models

*Egfr*^*f/f*^,(18) *Egfr*^*Wa5/+*^,(19) *Egfr*^*Dsk5/+*^,(20) and *Col 3.6-Cre*(21) mouse strains were generated as described previously. *Egfr*^*f/f*^ has two loxP sites flanking exon 3 of the EGFR. Deletion of exon 3 introduced a frameshift resulting in two stop codons in exon 4 and early termination of translation. *Egfr*^*Wa5/+*^ mice on a 129S1/SvImJ background were generated by breeding *Egfr*^*Wa5/+*^ and wild-type mice, and they were identified by their wavy hair appearance. *Egfr*^*Dsk5/+*^ mice on a 129S1/SvImJ background were obtained by breeding *Egfr*^*Dsk5/+*^ and wild-type mice. They were identified by wavy hair, hyperpigmented footpads, and long nails. To generate *Col-Cre Egfr*^*f/f*^ mice, we bred *Col 3.6-Cre* mice with *Egfr*^*f/f*^ mice to obtain *Col-Cre Egfr*^*f/+*^ mice. These mice then were backcrossed with *Egfr*^*f/f*^ mice to generate *Col-Cre Egfr*^*f/f*^ mice and their wild-type siblings. To generate *Col-Cre Egfr*^*Wa5/f*^ mice, we bred *Col 3.6-Cre* mice with *Egfr*^*Wa5/+*^ mice to obtain *Col-Cre Egfr*^*Wa5/+*^ mice. These mice then were crossed with *Egfr*^*f/f*^ mice to generate *Col-Cre Egfr*^*Wa5/f*^ mice and their siblings, *Col-Cre Egfr*^*f/+*^, *Egfr*^*Wa5/f*^, and *Egfr*^*f/+*^. *Col-Cre Egfr*^*Wa5/f*^ mice were identified by their wavy coat appearance and polymerase chain reaction (PCR) genotyping of the *Cre* gene using primers 5'-GAG TGA TGA GGT TCG CAA GA-3' and 5'-CTA CAC CAG AGA CGG AAA TC-3'. All work with animals was approved by the Institutional Animal Care and Use Committee (IACUC) at the University of Medicine and Dentistry of New Jersey and the University of Pennsylvania.

### EGFR inhibitor injection in mice

Four groups of 1-month-old female BALB/cAnNCr mice (National Cancer Institute at Frederick, Frederick, MD, USA) with 10 mice per group were treated with one of the following regimes: (1) 0.05% Tween-80, (2) 100 mg/kg of gefitinib dissolved in 0.05% Tween-80, (3) 0.5% methylcellulose, or (4) 50 mg/kg of erlotinib dissolved in 0.5% methylcellulose. All mice were treated daily with oral gavage for a total of 40 days before hind limbs were harvested for analysis. There was no significant difference in body weight gain between control and treated groups. There were no signs of dehydration, lethargy, or ataxia in any treatment group.

### Peripheral quantitative computed tomography (pQCT) analysis

The total and trabecular bone mineral density (BMD) values of the proximal tibias were evaluated ex vivo using an XCT Research SA (Stratec Medizintechnik, Pforzheim, Germany). A scout scan was run for a length of 10 mm. The pQCT scan was initiated 1.4 mm distal from the proximal epiphysis. The scan is 1 mm thick with a voxel size of 90 µm. Using an iterative algorithm, soft tissue (density below 223 mg/cm^3^) was removed automatically. The density of the remaining bone is reported as total density (mg/cm^3^). The outer 55% of the bone was peeled away in a concentric fashion to determine trabecular density (mg/cm^3^). Cortical BMD was measured in the femur. The femur length was determined after a 2D scout scan was run for the full length of the bone. The pQCT scan was initiated at the mid-diaphysis of the femur. The scan is 1 mm thick with a voxel size of 90 µm. Using an iterative algorithm, tissue with density below 500 mg/cm^3^ was removed automatically. The density of the remaining bone is reported as cortical density (mg/cm^3^).

### Micro–computed tomography (µCT) measurement

The femurs and tibias were subjected to ex vivo µCT analyses (Skyscan 1172 High-Resolution µCT; Skyscan, Antwerp, Belgium). To study the trabecular architecture of the proximal tibial metaphysis, a total of 160 slices with 5-µm resolution corresponding to the volume from 0.3 to 1.1 mm below the growth plate were reconstructed and analyzed using 3D analysis. To study the cortical bone parameters of the femoral midshaft, a total of 160 slices with 5-µm resolution corresponding to the volume from 2.2 to 3 mm below the growth plate were reconstructed and analyzed using 2D analysis.

### Trabecular bone histomorphometry

Mice were injected subcutaneously with 25 mg/kg of calcein (Sigma-Aldrich, St Louis, MO, USA) at 9 and 2 days before necropsy for dynamic histomorphometric measurements. Tibias were dissected and processed for methyl methacrylate embedding. Then 5-µm longitudinal sections were cut using a Polycut-S motorized microtome (Reichert, Heidelberg, Germany) and stained with Goldner's trichrome. Unstained 10-µm sections were used for dynamic measurements. Histomorphometric measurements were performed in the proximal tibial metaphyses in the area between 0.25 and 1.75 mm below the growth plate using the OsteoMeasure Analysis System (OsteoMetrics, Inc., Decatur, GA, USA). The primary indices include the total tissue area (TV), trabecular bone perimeter (BS), trabecular bone area (BV), osteoblast surface, osteoblast number, osteoclast surface, osteoclast number, osteoid surface, single- and double-labeled surface, and interlabel width. The percentage of osteoclast surface (OcS/BS), osteoid surface (OS/BS), osteoblast surface (ObS/BS), mineralizing surface (MS/BS), mineral apposition rate (MAR, µm/d), and surface-referent bone-formation rate (BFR/BS, µm^3^/µm^2^/yr) were calculated as described by Parfitt and colleagues.(22)

### Mouse bone marrow osteoblastic culture and colony-forming unit–fibroblast (CFU-F) assay

Bone marrow cells were flushed from femurs and tibias of 3-month-old wild-type and *Col-Cre Egfr*^*Wa5/f*^ mice, filtered, and seeded at 3 × 10^6^ cells per 35-mm dish for osteoblastic culture and at 3 × 10^6^ cells per T-25 flask for CFU-F assay in α minimum essential medium (α-MEM) supplemented with 15% fetal bovine serum (FBS), 1% glutamine, 0.1% β-mercaptoethanol, 100 IU/mL of penicillin, and 100 µg/mL of streptomycin. To obtained osteoblastic culture, the medium were changed on day 5 with the addition of 50 µg/mL of l-ascorbic acid. On day 12, cells were treated with EGF (50 ng/mL) and harvested at the indicated times for Western blot detection of total extracellular signal–regulated kinases (ERKs) and phosphorylated ERKs. The antibodies were purchased from Santa Cruz Biotechnology (Santa Cruz, CA, USA). For CFU-F assays, cells were fixed and stained with methyl violet on day 10. The number and diameter of colonies (each containing more than 20 cells) were counted and measured microscopically.

### Urinary deoxypyridinoline (DPD) analysis

Mice were housed in metabolic cages overnight, and urine samples were collected the next morning. The DPD and creatinine concentrations in the urine were determined by MicroVue DPD and Creatinine ELISA kits (Quidel, San Diego, CA, USA), respectively.

### Statistical analysis

Because the relationship between bone structural and histomorphometric parameters and age is not linear, the effect of genotype on pQCT, µCT, and histomorphometric data was analyzed by independent Student's *t* test assuming equal variances at each age group. For all statistical analyses, a value of *p* < .05 was considered significant. Animal number per group varied from 8 to 16. All data are expressed as mean ± SEM.

## Results

### *Col-Cre Egfr*^*f/f*^ mice do not exhibit a bone phenotype

As a first step to examine the role of EGFR signaling in bone development and remodeling, we bred *Col-Cre* and *Egfr*^*f/f*^ mice to generate preosteoblast and osteoblast-specific *Egfr* knockout mice, *Col-Cre Egfr*^*f/f*^. *Col 3.6-Cre* contains a 3.6-kb rat α1(I) collagen promoter–driven *Cre* and targets preosteoblasts and osteoblasts.(21) It has been used successfully to knock down preosteoblast/osteoblast expression in a number of mouse models. *Egfr*^*f/f*^ mice were used previously to generate *Egfr* null mice by crossing with mice carrying *EIIa-Cre*(18) and skin-specific *Egfr* knockout by crossing with *K14-Cre* transgenic mice.(23) To our surprise, *Col-Cre Egfr*^*f/f*^ mice did not exhibit any bone phenotype at 1 and 3 months of age in both genders compared with their wild-type siblings (*Col-Cre Egfr*^*f/+*^, *Egfr*^*f/+*^, and *Egfr*^*f/f*^; Supplemental Table S1). Genotyping *Egfr* alleles in mouse calvarial osteoblastic cells harvested from these mouse pups revealed that *Cre* expression did not completely convert *Egfr*^*f*^ to *Egfr*^*Δ*^, resulting in significant amounts of *Egfr*^*f*^ available to express EGFR (Supplemental Fig. S1).

### Trabecular bone phenotype of *Col-Cre Egfr*^*Wa5/f*^

**Fig. 1:**
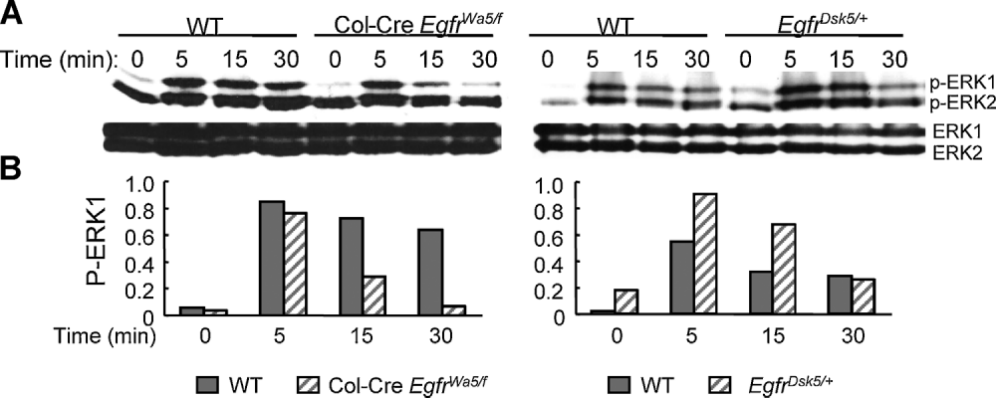
Mouse bone marrow osteoblastic cells derived from *Col-Cre Egfr^Wa5/f^* mice exhibit decreased ERK phosphorylation in response to EGF, whereas those derived from *Egfr^Dsk5/+^* mice have elevated ERK phosphorylation. (*A*) Western blots of phosphorylated ERK1/2 (*upper panel*) and total ERK1/2 (*bottom panel*). Cells were harvested at the indicated times after 50 ng/mL of EGF treatment. (*B*) The p-ERK1 level was quantified and normalized to total ERK1.

To overcome the preceding problem, we introduced the antimorphic *Wa5* allele into this system and generated *Col-Cre Egfr*^*Wa5/f*^ mice. *Wa5* has a single missense mutation Asp833Gly in the highly conserved DFG domain of EGFR kinase catalytic loop and codes for a kinase-dead dominant-negative receptor.(19) Homozygous mice are embryonic lethal, but heterozygous mice are viable and do not exhibit any bone phenotype at 1 and 3 months of age in both genders (Supplemental Table S2). We surmised that the presence of the *Wa5* allele would further reduce the remaining EGFR activity and result in a more complete knockdown of EGFR activity in preosteoblast/osteoblast lineage cells. To confirm this, we cultured bone marrow osteoblastic cells and treated them with EGF for 5, 15, and 30 minutes. Since Ras-Raf-MAP-kinase is the major EGFR downstream signaling pathway,(1) we performed Western blot analysis to detect the phosphorylation of ERKs. As shown in Fig. 1, EGF treatment stimulated a strong and sustained increase in the amounts of phosphorylated ERK1 in cells derived from wild-type mice, whereas it only induced ERK1 phosphorylation at 5 minutes in cells derived from *Col-Cre Egfr*^*Wa5/f*^ mice, and this induction was quickly diminished at 15 and 30 minutes (Fig. 1). Since, in the following studies, the bone phenotype did not differ between the three littermate control groups (*Col-Cre Egfr*^*f/+*^, *Egfr*^*Wa5/f*^, and *Egfr*^*f/+*^), all comparisons presented here are between wild-type control groups (*Col-Cre Egfr*^*f/+*^ and *Egfr*^*f/+*^) and *Col-Cre Egfr*^*Wa5/f*^ mice. Mice among all four groups have comparable body weight at all ages.

**Fig. 2:**
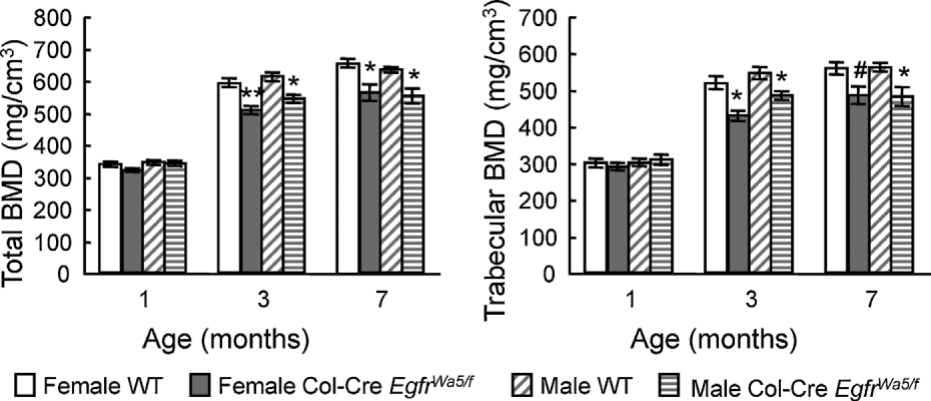
pQCT measurement of total and trabecular BMD of the proximal tibias of *Col-Cre Egfr^Wa5/f^* mice and their wild-type (WT) siblings at 1, 3, and 7 months of age. **p < .001; *p < .01; ^#^*p* < .05.

**Fig. 3:**
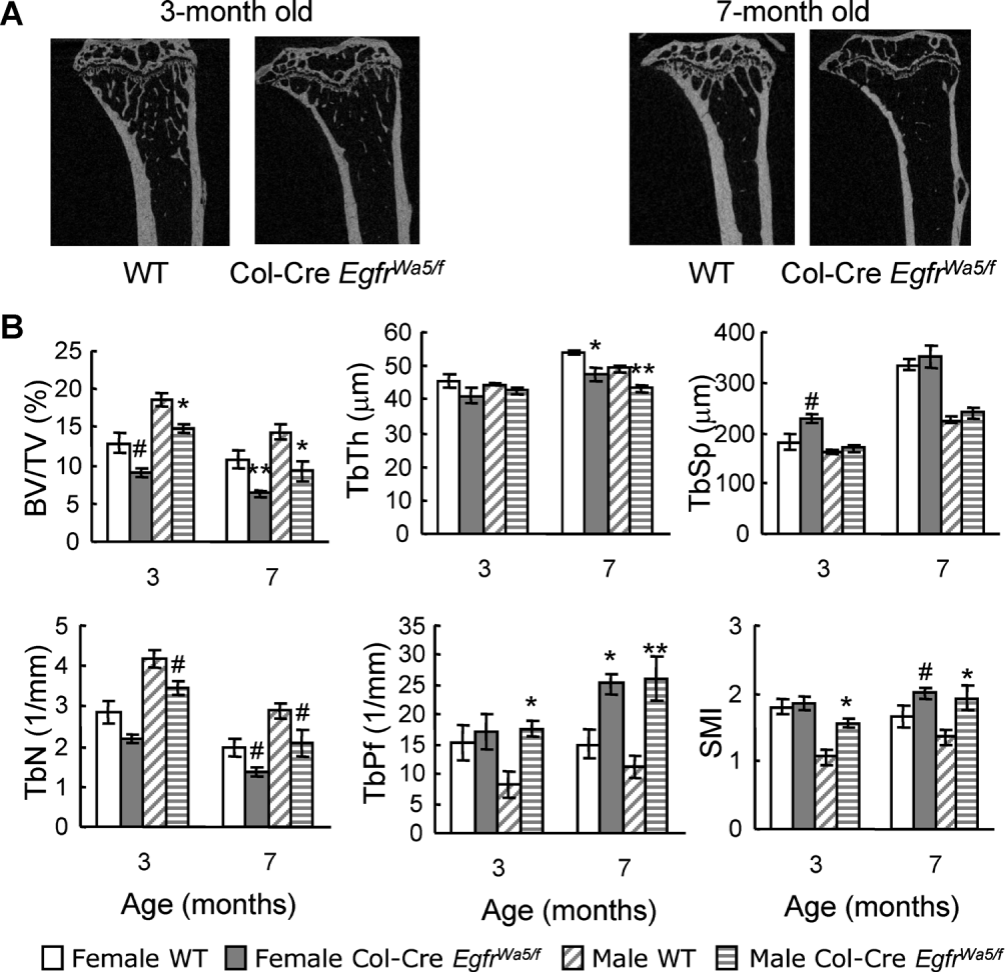
µCT scanning reveals that *Col-Cre Egfr^Wa5/f^* mice are osteopenic. (*A*) µCT images of longitudinal sections of proximal tibias of 3- and 7-month-old *Col-Cre Egfr^Wa5/f^* and their wild-type (WT) siblings. (*B*) Structural parameters of trabecular bone in the proximal tibias. BV/TV = trabecular bone volume/tissue volume; Tb.Th = trabecular thickness; Tb.Sp = trabecular separation; Tb.N = trabecular number; Tb.Pf = trabecular pattern factor; SMI = structure model index. **p < .001; *p < .01; ^#^*p* < .05.

We first performed pQCT analysis to measure the total and trabecular BMD values of the proximal tibias. As shown in Fig. 2, there was no change in both BMD values between wild-type and *Col-Cre Egfr*^*Wa5/f*^ mice at 1 month of age, when the skeleton is still immature and undergoing active modeling. However, when the skeleton matured at 3 months of age, there were significant decreases (12% to 17%) in total and trabecular BMD values in *Col-Cre Egfr*^*Wa5/f*^ mice compared with wild-type mice in both genders. A similar reduction in BMD value also was observed in mice at 7 months of age. Next, µCT was used to quantitatively access the structural parameters in the secondary spongiosa of the trabecular compartment in the same bones. Initial imaging suggested that there was significant trabecular bone loss in adult mice (Fig. 3*A*). Trabecular bone volume was considerably decreased in the *Col-Cre Egfr*^*Wa5/f*^ mice by 20% to 40% compared with control mice at 3 and 7 months of age (Fig. 3*B*). This was mainly due to the reduction in trabecular number (Tb.N, 17% to 32%). We also observed a significant decrease in trabecular thickness (Tb.Th, 10%) at 7 months of age and a nonsignificant decrease at 3 months. A significant increase in trabecular separation (Tb.Sp, 26%) was observed only in female mice at 3 months of age. Trabecular pattern factor (Tb.Pf) is an index for trabecular bone connectivity, and the structure model index (SMI) indicates the relative prevalence of rods and plates in the trabecular bone. The strong increases in both Tb.Pf and SMI in the *Col-Cre Egfr*^*Wa5/f*^ mice suggest that the structural integrity and mechanical strength of the trabecular bone were compromised in these EGFR-deficient animals.

**Fig. 4:**
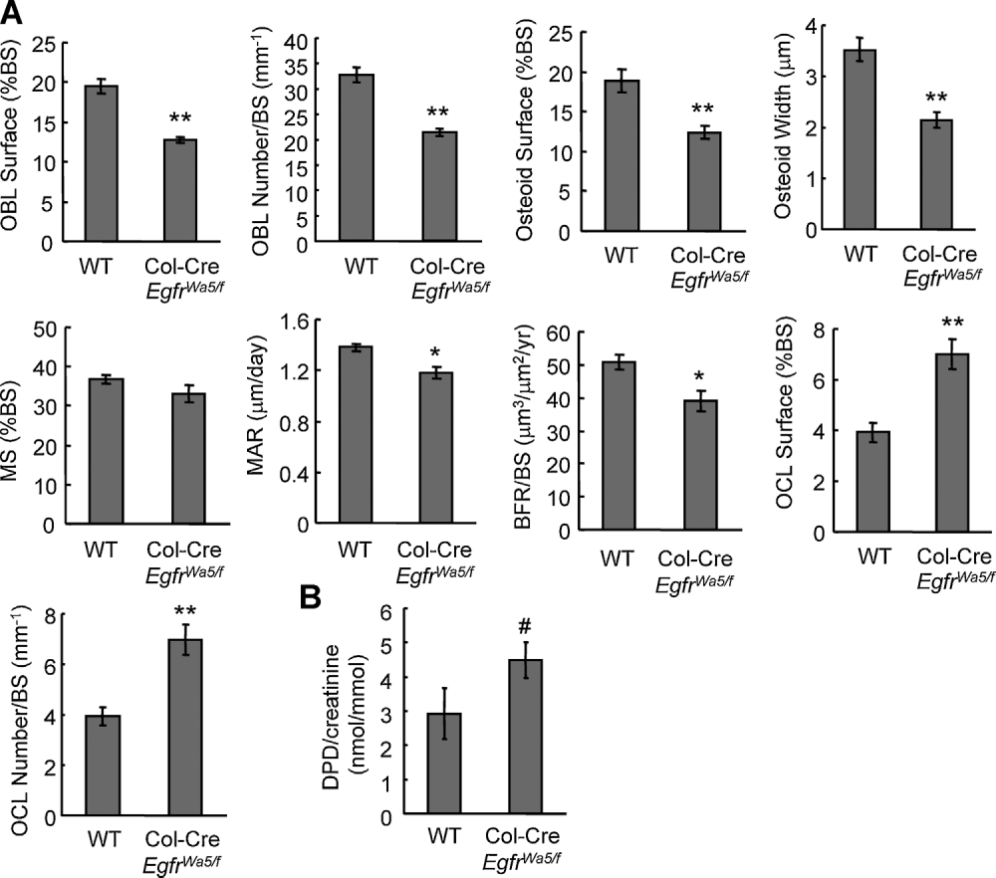
Bone formation and bone resorption are altered in *Col-Cre Egfr^Wa5/f^* mice. (*A*) Static and dynamic bone histomorphometry of the proximal tibias of 7-month-old female mice. OBL = osteoblast; OCL = osteoclast; MS = mineralizing surface; MAR = mineral apposition rate; BFR = bone-formation rate; BS = trabecular bone perimeter. **p < .001; *p < .01. (*B*) Urinary DPD assay. The DPD concentration was normalized to creatinine. ^#^*p* < .05.

Histomorphometric analyses of 7-month-old female *Col-Cre Egfr*^*Wa5/f*^ mice further revealed the cellular mechanism underlying the trabecular bone changes (Fig. 4*A*). Compared with wild-type mice, these mice exhibited dramatic decreases in osteoblast surface (35%) and osteoblast number (35%), along with similar decreases in osteoid surface (34%) and osteoid width (39%). Furthermore, MAR and BFR were reduced about 15% and 24%, respectively, whereas mineralizing surface (MS) remained unaffected. In addition, there were marked increases in the osteoclast surface (78%) and osteoclast number (78%). The increase of urinary DPD excretion in *Col-Cre Egfr*^*Wa5/f*^ mice further confirms the increase of bone resorption (Fig. 4*B*). The combination of the decrease in bone formation and the increase in bone resorption resulted in the dramatic loss of trabecular bone volume and microstructural deterioration in the *Col-Cre Egfr*^*Wa5/f*^ mice (Fig. 3).

### Cortical bone phenotype of *Col-Cre Egfr*^*Wa5/f*^

To assess the cortical bone phenotype of the *Col-Cre Egfr*^*Wa5/f*^ mice, the femoral midshaft was examined by pQCT. In comparison with wild-type mice, the femurs of the *Col-Cre Egfr*^*Wa5/f*^ mice are 3% to 5% shorter in length in both genders at 3 and 7 months of age (Table 1). While there were insignificant decreases in cortical BMD, cortical thickness, and endosteal perimeter, the cortical area and periosteal perimeter were reduced significantly by about 11% and 7%, respectively, in 3-month-old *Col-Cre Egfr*^*Wa5/f*^ mice. Furthermore, µCT analysis of male mice confirmed these results and demonstrated significant decreases in cortical area (18%, *p* < .001), cortical thickness (14%, *p* < .001), periosteal perimeter (7%, *p* < .01), and endosteal perimeter (7%, *p* = .05) compared with wild-type mice owing to the high resolution of µCT (data not shown). No change was observed in cortical bone porosity (data not shown). At 7 months of age, similar results were also obtained in mice of both genders using both pQCT and µCT (Table 1 and data not shown). Taken together, these data suggest that deficiency in osteoblastic EGFR activity affects cortical bone remodeling and results in smaller and thinner femurs. Moreover, µCT images also revealed smaller and thinner cortical bone in the tibial midshaft of *Col-Cre Egfr*^*Wa5/f*^ mice (Fig. 3*A*).

**Table 1:** Cortical Parameters of the Femoral Midshaft of *Col-Cre Egfr*^*Wa5/f*^ Mice Measured by pQCT

	Female		Male	
		
	Wild type	*Col-Cre Egfr*^*Wa5/f*^	Wild type	*Col-Cre Egfr*^*Wa5/f*^
3 months				
Cortical BMD (mg/cm^3^)	1073.8 ± 7.6	1059.7 ± 18.7	1111.2 ± 9.9	1098.9 ± 10.1
Cortical area (mm^2^)	1.22 ± 0.02	1.09 ± 0.03^a^	1.58 ± 0.04	1.42 ± 0.03^a^
Cortical thickness (mm)	0.403 ± 0.008	0.384 ± 0.007	0.440 ± 0.007	0.431 ± 0.009
Periosteal perimeter (mm)	4.30 ± 0.04	4.04 ± 0.08^a^	4.98 ± 0.06	4.65 ± 0.06^a^
Endosteal perimeter (mm)	1.77 ± 0.08	1.63 ± 0.08	2.21 ± 0.06	1.94 ± 0.09^b^
Length (cm)	1.57 ± 0.012	1.53 ± 0.02^b^	1.62 ± 0.01	1.56 ± 0.01^a^
7 months				
Cortical BMD (mg/cm^3^)	1196.2 ± 9.5	1153.2 ± 18.6	1195.0 ± 10.5	1139.9 ± 31.4
Cortical area (mm^2^)	1.39 ± 0.04	1.22 ± 0.04^a^	1.65 ± 0.03	1.51 ± 0.07
Cortical thickness (mm)	0.451 ± 0.009	0.429 ± 0.016	0.476 ± 0.010	0.457 ± 0.021
Periosteal perimeter (mm)	4.50 ± 0.07	4.21 ± 0.07^a^	4.97 ± 0.05	4.78 ± 0.11
Endosteal perimeter (mm)	1.67 ± 0.08	1.51 ± 0.12	1.98 ± 0.08	1.91 ± 0.17
Length (cm)	1.61 ± 0.02	1.53 ± 0.02^b^	1.64 ± 0.01	1.55 ± 0.01^a^

a *p* < .005

b *p* < .05 versus age- and sex- matched wild-type mice.

### *Col-Cre Egfr*^*Wa5/*f^ mice have fewer bone marrow mesenchymal stem cells (MSCs) and osteoprogenitors

**Fig. 5:**
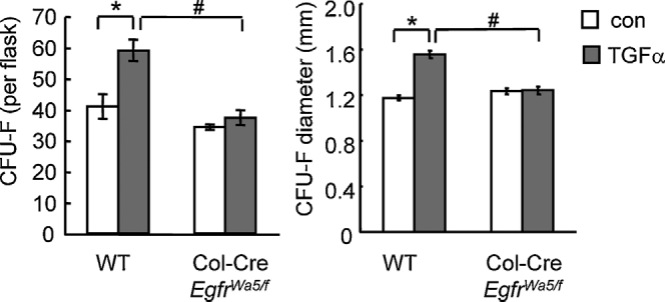
*Col-Cre Egfr^Wa5/f^* mice have fewer MSCs and committed osteoprogenitors. Bone marrow cells were cultured in growth medium without (con) or with TGF-α (20 ng/mL) for CFU-F assay. The number and size of CFU-F colonies were counted microscopically. *p < .05; ^#^*p* < .01.

Histomorphometry data revealed that *Col-Cre Egfr*^*Wa5/f*^ mice have less osteoblast surface, fewer osteoblast number, and less osteoid surface, implying that bone marrow osteoprogenitors might be affected in these mice. To analyze the mechanism, we performed CFU-F assays to measure the number and proliferative capacity of bone marrow MSCs and committed osteoprogenitors from these mice. Specifically, we cultured bone marrow cells in growth medium and measured the number and size of CFU-F colonies. As shown in Fig. 5, we did not detect any significant decreases in the number and diameter of colonies from *Col-Cre Egfr*^*Wa5/f*^ mice in normal culture conditions. Interestingly, when TGF-α (20 ng/mL) was added to the cultures, there were significant increases in colony number and size in cells from wild-type mice, indicating that normal culture medium lacks sufficient amounts of the EGF ligands required for optimal survival and proliferation of MSCs and osteoprogenitors on the plastic surface. This is consistent with previous reports that addition of EGF increases the CFU-F numbers when human(24) and mouse MSCs(25) were cultured in medium supplemented with FBS. However, similar increases in CFU-F number and size were not observed in cells derived from *Col-Cre Egfr*^*Wa5/f*^ mice, resulting significantly lower CFU-F number and smaller colony size in cells from these mice compared with those from wild-type mice. These data clearly suggest that these mice have fewer MSCs and osteoprogenitors and that the proliferative capacity of these cells is also reduced.

### EGFR inhibitors result in a bone phenotype similar to that of genetically EGFR-deficient mice

**Fig. 6:**
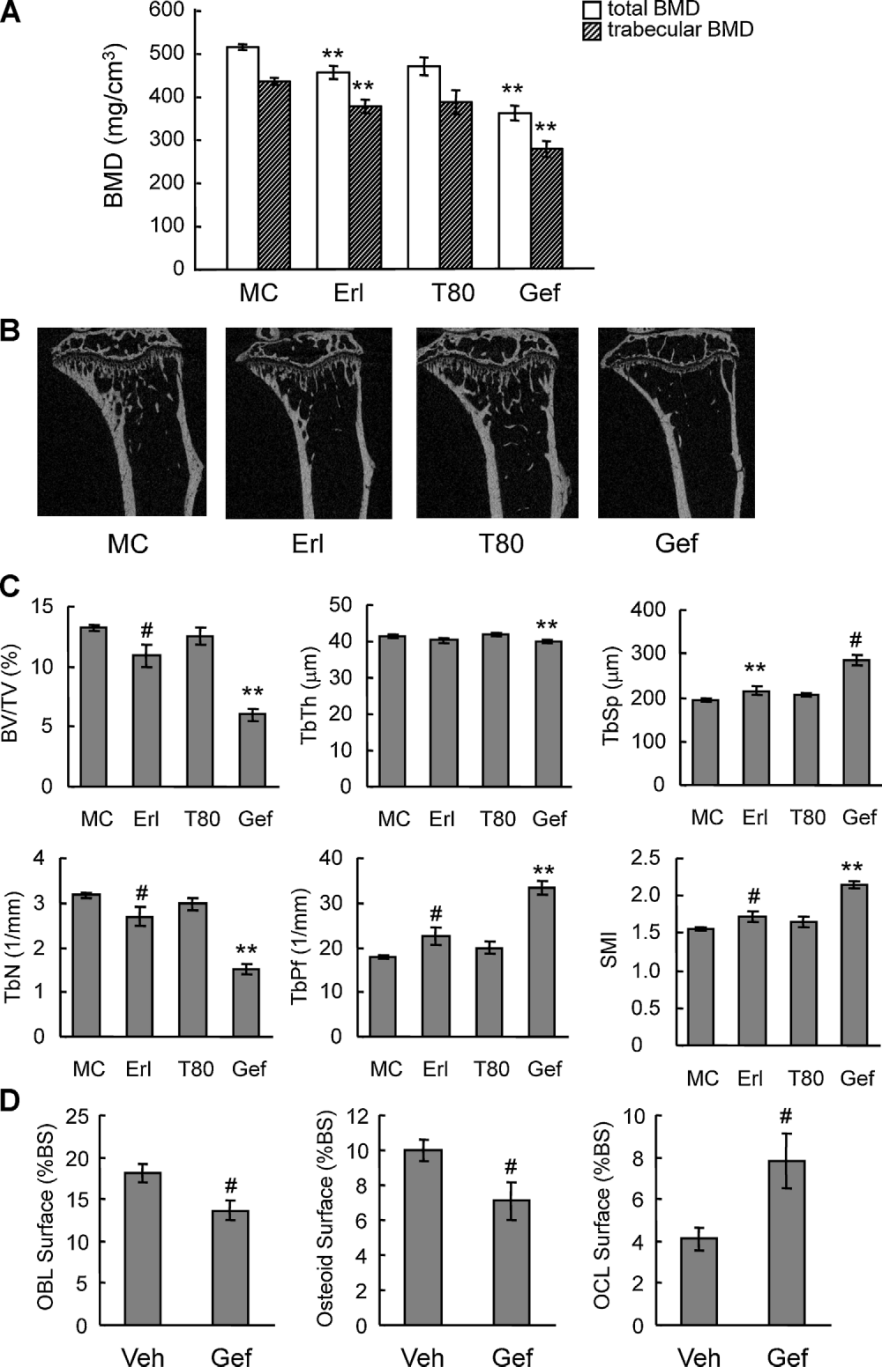
EGFR inhibitors decrease trabecular bone content. (*A*) pQCT measurement of total and trabecular BMD values of the proximal tibias of EGFR inhibitor–treated mice. MC = vehicle control for erlotinib treatment; Erl = 50 mg/kg/day erlotinib treatment; T80 = vehicle control for gefitinib treatment; Gef = 100 mg/kg/day gefitinib treatment. (*B*) µCT images of longitudinal sections of proximal tibias. (*C*) µCT analysis of proximal tibias of EGFR inhibitor–treated mice. (*D*) Static bone histomorphometric analysis in the same bone region. **p < .001; *p < .01; ^#^*p* < .05.

Administration of EGFR inhibitors gefitinib or erlotinib into 1-month-old wild-type mice for 6 weeks produced a significant reduction in trabecular bone content compared with the corresponding vehicle-treated mice. pQCT measurements of the proximal tibial region showed (Fig. 6*A*) that total and trabecular BMD decreased by 11% to 13% in the erlotinib-treated animals and 23% to 28% in the gefitinib-treated animals compared with their respective vehicle controls. µCT images confirmed a more dramatic loss of trabecular bone in the gefitinib-treated mice (Fig. 6*B*). Quantitative analysis showed that trabecular bone content (BV/TV) decreased by 52% in gefitinib-treated mice, which was attributed to a large decrease in Tb.N (49%) and a moderate decrease in Tb.Th (5%) along with a notable increase in Tb.Sp (38%; Fig. 6*C*). In elotinib-treated mice, the loss of bone content was relatively moderate with an 18% decrease in BV/TV, accompanied by an 11% increase in Tb.Sp and a 15% decrease in Tb.Th. Significant increases in Tb.Pf and SMI with both inhibitors indicate a decrease in structural integrity and deterioration in trabecular microarchitecture. The more dramatic effects observed in gefitinib-treated mice are probably due to the high dosage of gefitinib (100 mg/kg/day versus 50 mg/kg/day for erlotinib). We also noted similar changes in trabecular bone structure in 3-month-old EGFR inhibitor–treated mice (data not shown).

Similar to *Col-Cre Egfr*^*Wa5/f*^ mice, the trabecular bone loss in gefitinib-treated mice is due to decreases in osteoblast (25%) and osteoid surfaces (29%) and a dramatic increase in osteoclast number (90%; Fig. 6*D*). Effects of the inhibitors also were apparent in the femoral cortical compartment (Table 2), where cortical BMD, cortical area and thickness, and periosteal and endosteal perimeters exhibited a decreasing trend in inhibitor-treated mice. Statistical significance was reached only for cortical BMD and thickness of the gefitinib-treated mice.

**Table 2:** Cortical Bone Parameters of the Femoral Midshaft of EGFR Inhibitor–Treated Mice Measured by pQCT

	MC	Erlotinib	T80	Gefitinib
Cortical BMD (mg/cm^3^)	1002.2 ± 10.2	985.9 ± 13.3	1008.9 ± 8.1	964.8 ± 10.1^a^
Cortical area (mm^2^)	0.79 ± 0.01	0.76 ± 0.02	0.78 ± 0.02	0.72 ± 0.03
Cortical thickness (mm)	0.179 ± 1.3	0.176 ± 0.002	0.177 ± 0.003	0.163 ± 0.003^a^
Periosteal perimeter (mm)	4.88 ± 0.04	4.76 ± 0.06	4.82 ± 0.07	4.79 ± 0.08
Endosteal perimeter (mm)	3.50 ± 0.04	3.41 ± 0.04	3.44 ± 0.06	3.54 ± 0.06

MC = vehicle control for erlotinib treatment; Erlotinib = 50 mg/kg/day; T80 = vehicle control for gefitinib treatment; Gefitinib = 100 mg/kg/day.

a *p* < .05 versus corresponding vehicle.

### The bone phenotype of *Egfr*^*Dsk5/+*^ mice with high EGFR activity

**Fig. 7:**
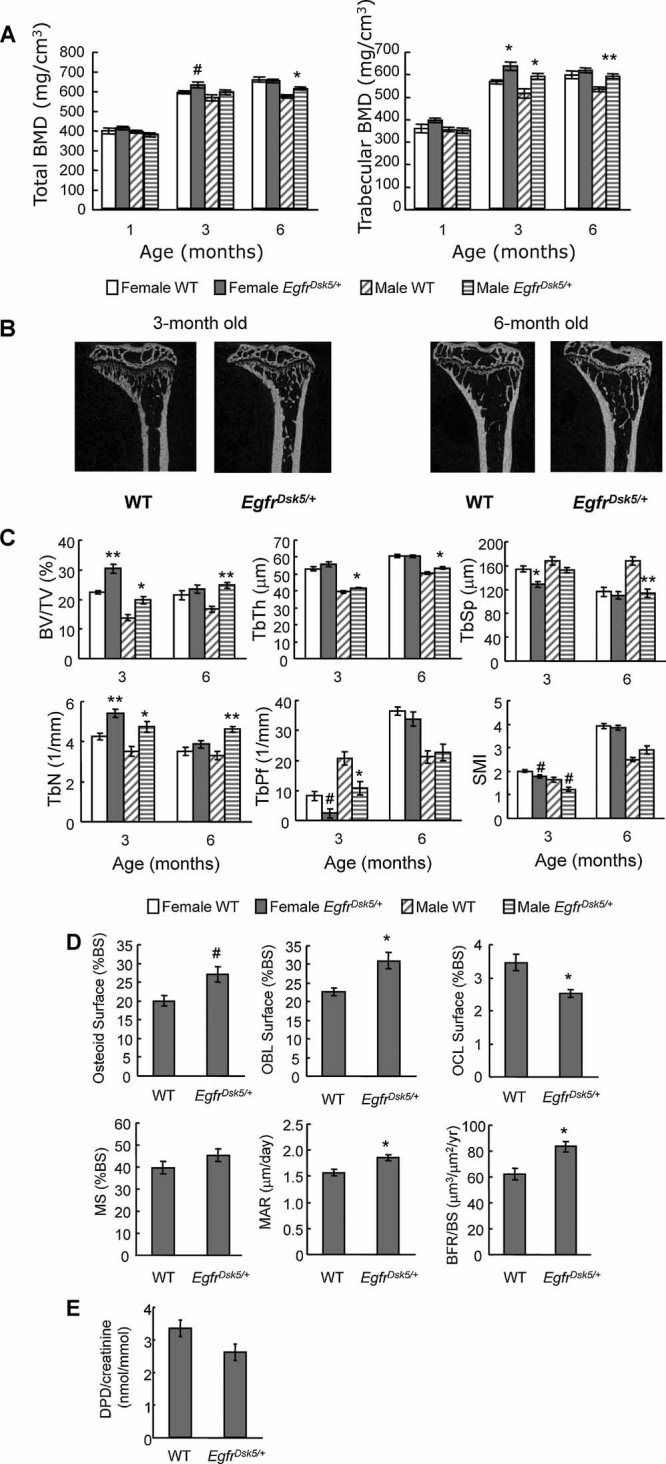
*Egfr^Dsk5/+^* mice with high EGFR activity have higher bone content. (*A*) pQCT analysis of total and trabecular BMD values of the proximal tibias of *Egfr^Dsk5/+^* mice and their wild-type siblings at 1, 3, and 6 months of age. (*B*) µCT images of longitudinal sections of proximal tibias. (*C*) µCT analysis of proximal tibias of *Egfr^Dsk5/+^* mice and their wild-type siblings. (*D*) Static and dynamic bone histomorphometric analyses of the proximal tibias of 3-month-old female *Egfr^Dsk5/+^* mice. (*E*) Urinary DPD assay. *p* = .059. **p < .001; *p < .01; ^#^*p* < .05.

*Dsk5* has a Leu863Gln mutation within a region of the kinase domain important for stabilization of the receptor activation loop, and therefore, the mutation is a gain-of-function allele that causes increased EGFR signaling.(20) Indeed, we observed an increased basal level of ERK phosphorylation in osteoblastic cells from these mice, and after EGF stimulation, the levels of phosphorylated ERKs were much higher than those in cells from wild-type mice (Fig. 1). Similar to *Col-Cre Egfr*^*Wa5/f*^ mice, *Egfr*^*Dsk5/+*^ mice showed no changes in BMD at 1 month of age (Fig. 7*A*). However, when their skeleton matured at 3 months of age, both female and male *Egfr*^*Dsk5/+*^ mice had significantly increased trabecular BMD values (12% and 15%, respectively; Fig. 7*A*), in contrast to the bone loss phenotype of *Col-Cre Egfr*^*Wa5/f*^ mice with low EGFR activity. The total BMD also was increased slightly, although it only achieved statistical significance in females. At 6 months of age, the difference in BMD was less apparent in the female mice, but male *Egfr*^*Dsk5/+*^ mice still maintained higher total (7%) and trabecular (11%) BMD values compared with their wild-type siblings.

µCT images confirmed the increase in the tibial trabecular area in *Egfr*^*Dsk5/+*^ mice (Fig. 7*B*). The increase in bone content (BV/TV) at 3 months of age (36% for female and 43% for male mice) was accompanied by a major increase in Tb.N (28% to 35%), a slight increase in Tb.Th (5%; Fig. 7*C*), and a moderate decrease in Tb.Sp (9% to 17%). The significant reductions in Tb.Pf and SMI indicate that trabecular microarchitecture was modified to have more connectivity and healthier platelike structures in these mice. These results are the opposite of the changes we observed with *Col-Cre Egfr*^*Wa5/f*^ mice. Consistent with the pQCT data, female *Egfr*^*Dsk5/+*^ mice did not exhibit a bone phenotype at the age of 6 months, but male mice still showed a remarkable increase in bone volume (49%) with similar changes in trabecular structural parameters.

Histologic analysis revealed that the increase in trabecular bone volume in *Egfr*^*Dsk5/+*^ mice was due to changes in both bone formation and bone resorption. In 3-month-old female *Egfr*^*Dsk5/+*^ mice, we observed a 37% increase in osteoblast surface, 35% increase in unmineralized osteoid surface, 14% increase in MS, 18% increase in MAR, and 34% increase in BFR compared with wild-type mice (Fig. 7*D*). Meanwhile, bone resorption was greatly decreased, with a 27% reduction in osteoclast surface (Fig. 7*D*) and a 22% decrease in urinary DPD concentration (*p* = .059; Fig. 7*E*). These results are all opposite to the changes we observed in the *Col-Cre Egfr*^*Wa5/f*^ mice.

There were significant changes in cortical bone structure at the femur midshaft in both male and female *Egfr*^*Dsk5/+*^ mice at 6 months of age (Table 3). We observed a significant increase in cortical BMD in the female group and a nonsignificant increase in the male group. This is due mainly to the 10% increase in the cortical thickness. We also observed significant decreases in both periosteal and endosteal perimeters (4% and 30%, respectively).

**Table 3:** Cortical Parameters of the Femoral Midshaft of *Egfr*^*Dsk5/+*^ Mice Measured by pQCT

	Female		Male	
		
	Wild type	*Egfr*^*Dsk5/+*^	Wild type	*Egfr*^*Dsk5/+*^
3 months				
Cortical BMD (mg/cm^3^)	1087.3 ± 8.8	1110.2 ± 8.1	1137.5 ± 12.3	1159.3 ± 11.5
Cortical area (mm^2^)	1.05 ± 0.03	1.07 ± 0.03	1.22 ± 0.07	1.24 ± 0.05
Cortical thickness (mm)	0.382 ± 0.009	0.412 ± 0.013	0.445 ± 0.013	0.493 ± 0.015^c^
Periosteal perimeter (mm)	3.95 ± 0.07	3.90 ± 0.06	4.14 ± 0.13	4.06 ± 0.09
Endosteal perimeter (mm)	1.55 ± 0.10	1.31 ± 0.11	1.44 ± 0.07	1.14 ± 0.05^b^
Length (cm)	1.52 ± 0.01	1.50 ± 0.01	1.56 ± 0.02	1.55 ± 0.01
6 months				
Cortical BMD (mg/ cm^3^)	1183.4 ± 9.0	1214.7 ± 10.1^c^	1189.5 ± 9.2	1211.2 ± 7.736
Cortical area (mm^2^)	1.34 ± 0.02	1.31 ± 0.01	1.34 ± 0.03	1.35 ± 0.03
Cortical thickness (mm)	0.444 ± 0.008	0.487 ± 0.018^b^	0.443 ± 0.009	0.505 ± 0.011^a^
Periosteal perimeter (mm)	4.42 ± 0.03	4.23 ± 0.02^a^	4.41 ± 0.05	4.27 ± 0.05^c^
Endosteal perimeter (mm)	1.63 ± 0.05	1.17 ± 0.13^b^	1.63 ± 0.07	1.10 ± 0.09^a^
Length (cm)	1.63 ± 0.01	1.62 ± 0.01	1.57 ± 0.01	1.60 ± 0.01^b^

a *p* < .001

b *p* < .01

c *p* < .05 versus age- and sex-matched wild-type mice.

## Discussion

EGFR is extremely critical for embryonic development and organogenesis, and therefore mice with systemic *Egfr* knockout are either embryonic lethal or die shortly after birth owing to placental defects and multiorgan abnormalities,(13) making it impossible to study the role of EGFR signaling in bone development and remodeling postnatally. Nevertheless, a bone phenotype was observed in several previous reports investigating either *Egfr* null mice at birth(14,15) or transgenic mice ubiquitously overexpressing its ligands, EGF(26) or BTC,(17) under the control of ubiquitous actin promoters. Moreover, our previous data found that *amphiregulin* null mice had less trabecular bone at 1 month of age.(5) These findings prompted us to study the in vivo role of EGFR in bone metabolism in adult animals. To our knowledge, this is the first report demonstrating that under physiologic conditions, abrogating EGFR activity in osteoblasts results in an osteopenic phenotype in adult mice of both sexes. Detailed analyses of bone structural parameters by µCT revealed that trabecular bone loss in *Col-Cre Egfr*^*Wa5/f*^ mice was due to decreases in trabecular number and size, accompanied by deterioration of microachitecture, as evidenced by increases in Tb.Pf and SMI. Furthermore, bone histomorphometric studies demonstrated a significant decrease in bone formation and an increase in bone resorption. Loss of EGFR activity also affected the femoral cortical structure and resulted in smaller and thinner cortical bone. Moreover, additional mouse models with either a decrease or an increase in EGFR activity (EGFR inhibitor–treated mice and *Egfr*^*Dsk5/+*^ mice, respectively) exhibited consistent bone phenotype changes. Taken together, these data indicate that EGFR activity mainly plays an anabolic role in bone development and remodeling.

To study the function of osteoblastic EGFR, we initially generated *Col-Cre Egfr*^*f/f*^ mice, but surprisingly, no bone abnormality was apparent in these mice. *Dermo1-Cre* targets embryonic condensed mesenchyme, from which chondrocytes and osteoblasts are derived.(27) Similarly, *dermo1-Cre Egfr*^*f/f*^ mice exhibit normal bones (data not shown). Genotyping *Egfr* alleles in mouse calvarial osteoblastic cells harvested from Col-Cre *Egfr*^*f/f*^ mice revealed that there are significant amounts of *Egfr*^*f*^ remaining. However, further decreasing this residual EGFR activity by introducing a dominant-negative *Wa5* allele into mice resulted in a strong bone phenotype, suggesting that EGFR activity needs to be reduced to a very low level to affect bone structures. EGFRs are expressed widely in cells of mesodermal and ectodermal origin but not in cells of hematopoietic origin.(28) While a complete knockdown of *Egfr* is embryonic lethal, decreasing EGFR activity to a certain degree seems to have only minor effects. For example, *Egfr*^+/–^ mice are viable and fertile and behave virtually the same as *Egfr*^+/+^ mice. In vitro, equimolar expression of *Egfr* and *Egfr*^*wa5*^ in CHO cells results in less than 10% of wild-type phosphorylation of the EGFR, but in vivo, the only significant phenotypes observed in *Egfr*^*Wa5/+*^ mice are open eyelids at birth and wavy coat.(19) We did not observe any trabecular or cortical bone abnormalities in *Egfr*^*Wa5/+*^ mice. Consistent with this, previous studies showed that total volumetric BMD, cross-sectional area, and cortical thickness in *Egfr*^*Wa5/+*^ femurs were the same as in wild-type siblings.(17)

Our conclusion that EGFR primarily plays an anabolic role in bone metabolism is in agreement with other studies. Previous data from our laboratory showed decreases in trabecular BMD and bone volume but no change in cortical bone parameters in *amphiregulin* knockout mice.(5) In humeri of *Egfr* null mice, there were fewer trabeculae than in wild-type mice at E18.5, and this phenomenon persisted until birth.(15) The most dramatic effect of ubiquitous overexpression of BTC in mice (BTC-Tg) is high cortical bone mass in femurs with increased cortical area and thickness owing to augmented endocortical bone apposition.(17) This effect depends on the EGFR because it was blocked in the *Egfr*^*Wa5*^ background. These data are consistent with our findings that EGFR activity is positively correlated with cortical bone size. However, BTC-Tg mice showed profound increases in cortical porosity, which was not observed in our mouse models. Another discrepancy was the reduction of the vertebral BMD in BTC-Tg mice. The femoral trabecular bone volume was higher only in female transgenic mice at 6 weeks of age, but not in the males, and this increase was not translated into higher trabecular BMD. The difference described earlier could be due to high nonphysiologic expression of BTC under the control of the chicken β-actin promoter in transgenic mice, and we believe that our models are more accurate in revealing the osteoblastic EGFR functions.

Histomorphometric analyses revealed decreased bone formation and increased bone resorption in the EGFR-deficient models and vice versa in the EGFR- augmented model. EGF-like ligands have direct actions on osteoblast proliferation and differentiation. EGF has been known for a long time as a mitogen for both UMR 106-01 cells, a rat osteosarcoma osteoblastic cell line, and primary calvarial osteoblastic cells.(29) In our past studies, we demonstrated that amphiregulin has strong proliferative effects on preosteoblasts and that EGFR signaling is important for normal growth of osteoblastic cells. Osteoblasts originate from mesenchymal stem cells, and EGF and HB-EGF are important growth factors for maintaining their growth in vitro.(3,4) In vitro experiments also showed that activation of EGFR strongly suppresses osteogenesis and particularly inhibits bone marker genes such as *alkaline phosphatase*, *type 1 collagen*, *BSP*, and *osteocalcin*(5,30,31) and osteoblastic-specific transcription factors *Runx2* and *osterix.*(6) Furthermore, studies of primary calvarial osteoblastic cultures from EGFR-deficient mice, such as *Egfr*^*-/-*^ mice, and humanized EGFR mice showed a decrease in osteoblast proliferation and an increase in mineralization.(16) Interestingly, we found that the number and size of CFU-F colonies from *Col-Cre Egfr*^*Wa5/f*^ mice were decreased in comparison with wild-type mice when EGF-like ligands were added to the culture medium, suggesting that these EGFR-defective mice has fewer osteoprogenitors and that these osteoprogenitors are less proliferative. Combining these in vitro and in vivo mouse models data together, we hypothesize that osteoblastic EGFR signaling maintains a pool of osteoprogenitors and keeps these cells at an undifferentiated stage for future osteogenesis stimulated by other factors.

To date, all in vitro data point out that EGFR signaling enhances bone resorption by osteoclasts, although it is still controversial whether EGF-like ligands act directly or indirectly on osteoclasts. It has long been demonstrated that EGF stimulates ^45^Ca release in fetal rat long bone cultures.(8,32) Gefitinib inhibits the ability of the MSC-like cell line HDS to support osteoclastogenesis by suppressing the expression of M-CSF and RANKL.(33) Studies from our laboratory found that osteoclasts do not bind [^125^I]EGF and do not respond to EGF treatment. Instead, EGF stimulates osteoclastogenesis in osteoblast/osteoclast cocultures by blocking the synthesis of OPG and increasing the amount of MCP-1 produced by osteoblasts.(10) However, Yi and colleagues recently demonstrated that mouse primary bone marrow osteoclast cultures do express EGFR protein, as detected by Western blot and immunofluorescence. Suppression of EGFR activity using an inhibitor or siRNA interfered with the RANKL signaling and subsequently reduced osteoclast differentiation.(34) Hence we were surprised to observe the negative correlation between EGFR activity and bone resorption in our mouse models. It is possible that in vitro experiments have used high doses of EGF and are more reminiscent of situations where cancer cells with high expression levels of EGF-like ligands result in osteolytic lesions in bone.(10,35) Under physiological conditions, the expression of EGF-like ligands in bone may be low, and therefore, activating osteoblastic EGFR activity actually decreases osteoclast formation and bone resorption. In addition, we found that there were no difference in the *Rankl*/*Opg* mRNA ratio in the femoral secondary spongiosa harvested from *Col-Cre Egfr*^*Wa5/f*^ and *Egfr*^*Dsk5/+*^ mice and their respective wild-type controls (data not shown). Further experiments are required to address this discrepancy between these in vitro and in vivo data.

In conclusion, our study demonstrates that osteoblastic EGFR signaling primarily plays an anabolic role in bone metabolism, implying its potential role in osteoporosis. This finding establishes EGFR as an important signaling molecule in bone development and remodeling and warrants further exploration of EGFR as an anabolic drug target. It would be of interest to study whether and how EGFR signaling engages other important bone signaling pathways, such as BMP, Notch, and Wnt.
