# Supplementary material for: Epidermal Growth Factor Receptor Plays an Anabolic Role in Bone Metabolism In Vivo
Source: J Bone Miner Res. 2010 Nov 18;26(5):1022–34. doi: 10.1002/jbmr.295 (PMC3179301; doi:10.1002/jbmr.295)
Supplement: Supplementary file 1 [file jbmr0026-1022-SD1.ppt]

## Slide 1
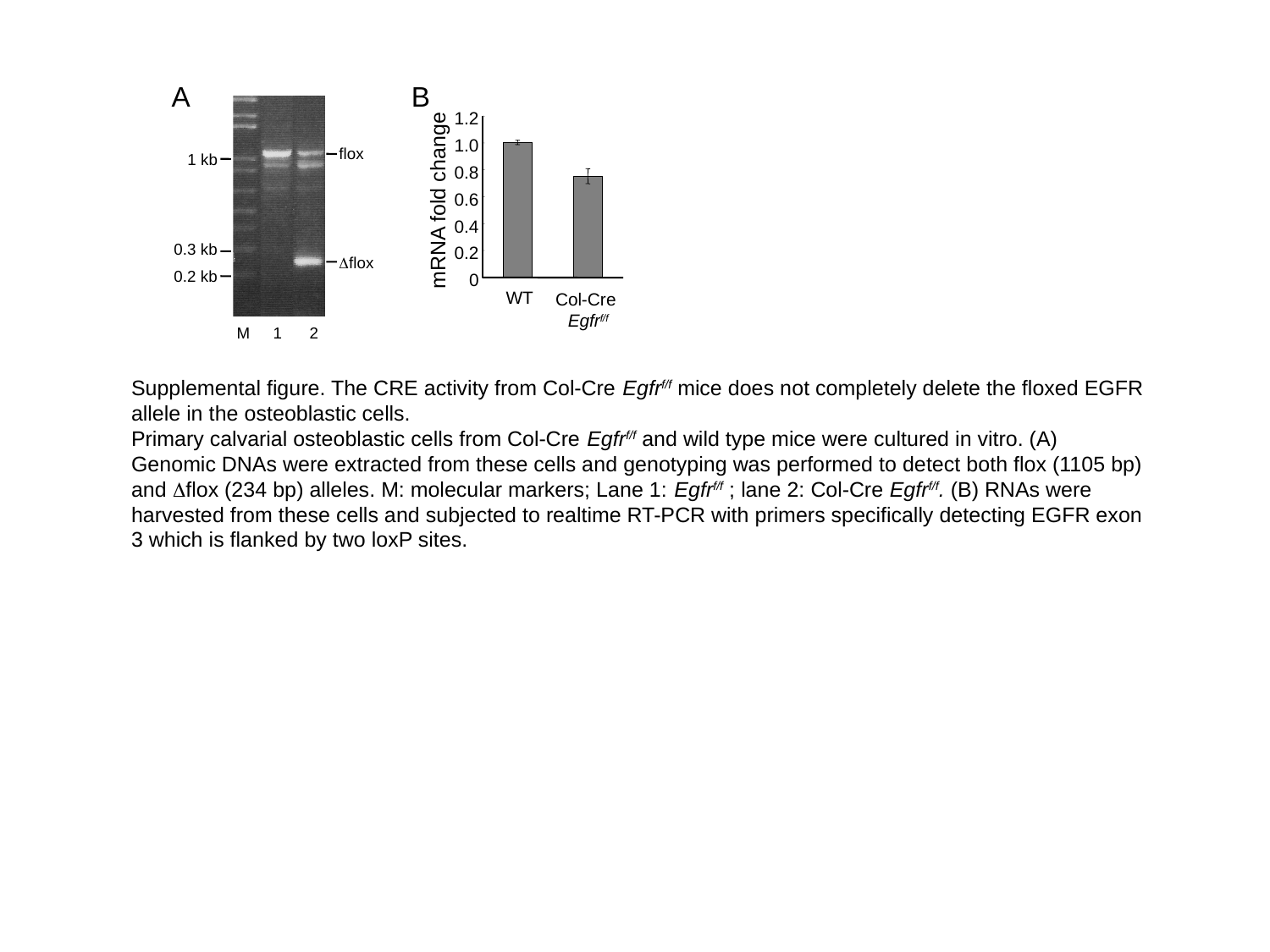

A
B
1.2
1.0
flox
1 kb
0.8
mRNA fold change
0.6
0.4
0.3 kb
0.2
Δflox
0.2 kb
0
WT
Col-Cre
Egfrf/f
M
2
1
Supplemental figure. The CRE activity from Col-Cre Egfrf/f mice does not completely delete the floxed EGFR allele in the osteoblastic cells.
Primary calvarial osteoblastic cells from Col-Cre Egfrf/f and wild type mice were cultured in vitro. (A) Genomic DNAs were extracted from these cells and genotyping was performed to detect both flox (1105 bp) and Δflox (234 bp) alleles. M: molecular markers; Lane 1: Egfrf/f ; lane 2: Col-Cre Egfrf/f. (B) RNAs were harvested from these cells and subjected to realtime RT-PCR with primers specifically detecting EGFR exon 3 which is flanked by two loxP sites.
