# Supplementary material for: Epidermal Growth Factor Receptor Plays an Anabolic Role in Bone Metabolism In Vivo
Source: J Bone Miner Res. 2010 Nov 18;26(5):1022–34. doi: 10.1002/jbmr.295 (PMC3179301; doi:10.1002/jbmr.295)
Supplement: Supplementary file 2 [file jbmr0026-1022-SD2.doc]

Supplemental table 1. Total and trabecular BMDs of proximal tibial bones from Col-Cre *Egfr^f/f^* mice measured by pQCT.

|  | **Female** | | **Male** | |
| --- | --- | --- | --- | --- |
|  | **Wild type** | **Col-Cre *Egfr^f/f^*** | **Wild type** | **Col-Cre *Egfr^f/f^*** |
| 1 month |  |  |  |  |
| Total BMD (mg/cm^3^) | 337.0 ± 14.0 | 330.8 ± 7.9 | 359.0 ± 9.7 | 352.6 ± 8.1 |
| Trabecular BMD (mg/cm^3^) | 317.1 ± 16.8 | 312.1 ± 10.7 | 345.4 ± 13.3 | 340.4 ± 14.0 |
| 3 months |  |  |  |  |
| Total BMD (mg/cm^3^) | 541.9 ± 12.0 | 523.8 ± 27.9 | 580.5 ± 14.9 | 584.9 ± 19.6 |
| Trabecular BMD (mg/cm^3^) | 488.8 ± 15.2 | 449.1 ± 33.4 | 529.1 ± 17.6 | 543.6 ± 19.6 |
