# Supplementary material for: Epidermal Growth Factor Receptor Plays an Anabolic Role in Bone Metabolism In Vivo
Source: J Bone Miner Res. 2010 Nov 18;26(5):1022–34. doi: 10.1002/jbmr.295 (PMC3179301; doi:10.1002/jbmr.295)
Supplement: Supplementary file 3 [file jbmr0026-1022-SD3.doc]

Supplemental table 2. Total and trabecular BMDs of proximal tibial bones from *Egfr^Wa5/+^* mice measured by pQCT.

|  | **Female** | | **Male** | |
| --- | --- | --- | --- | --- |
|  | **Wild type** | ***Egfr^Wa5/+^*** | **Wild type** | ***Egfr^Wa5/+^*** |
| 1 month |  |  |  |  |
| Total BMD (mg/cm^3^) | 394.1 ± 9.6 | 369.4 ± 7.1 | 347.9 ± 7.1 | 340.9 ± 21.3 |
| Trabecular BMD (mg/cm^3^) | 370.5 ± 10.4 | 346.4 ± 7.9 | 318.5 ± 5.7 | 312.2 ± 22.2 |
| 3 months |  |  |  |  |
| Total BMD (mg/cm^3^) | 590.0 ± 17.0 | 605.7 ± 8.6 | 570.2 ± 21.9 | 560.4 ± 13.0 |
| Trabecular BMD (mg/cm^3^) | 563.1 ± 25.8 | 586.2 ± 8.8 | 521.1 ± 25.8 | 523.9 ± 13.7 |
